# Projected Streamflow in the Kurau River Basin of Western Malaysia under Future Climate Scenarios

**DOI:** 10.1038/s41598-020-65114-w

**Published:** 2020-05-20

**Authors:** Muhammad Nasir Mohd Adib, Md Kamal Rowshon, Md Abdul Mojid, Ismail Habibu

**Affiliations:** 1 Department of Biological and Agricultural Engineering, Faculty of Engineering, Universiti Putra Malaysia, 43400 UPM Serdang, Selangor DE Malaysia; 2 Faculty of Agricultural and Engineering Technology, Bangladesh Agricultural University, Mymensingh 2202, Bangladesh; 3 Department of Agricultural and Bio-Resources Engineering, Ahmadu Bello University, Zaria, Nigeria

**Keywords:** Projection and prediction, Hydrology

## Abstract

Climate change-induced spatial and temporal variability of stremflow has significant implications for hydrological processes and water supplies at basin scale. This study investigated the impacts of climate change on streamflow of the Kurau River Basin in Malaysia using a Climate-Smart Decision Support System (CSDSS) to predict future climate sequences. For this, we used 25 reliazations consisting from 10 Global Climate Models (GCMs) and three IPCC Representative Concentration Pathways (RCP4.5, RCP6.0 and RCP8.5). The generated climate sequences were used as input to Soil and Water Assessment Tool (SWAT) to simulate projected changes in hydrological processes in the basin over the period 2021–2080. The model performed fairly well for the Kurau River Basin, with coefficient of determination (R^2^), Nash-Sutcliffe Efficiency (NSE) and Percent Bias (PBIAS) of 0.65, 0.65 and –3.0, respectively for calibration period (1981–1998) and 0.60, 0.59 and −4.6, respectively for validation period (1996–2005). Future projections over 2021–2080 period show an increase in rainfall during August to January (relatively wet season, called the main irrigation season) but a decrease in rainfall during February to July (relatively dry season, called the off season). Temperature projections show increase in both the maximum and minimum temperatures under the three RCP scenarios, with a maximum increase of 2.5 °C by 2021–2080 relative to baseline period of 1976–2005 under RCP8.5 scenario. The model predicted reduced streamflow under all RCP scenarios compared to the baseline period. Compared to 2021–2050 period, the projected streamflow will be higher during 2051–2080 period by 1.5 m^3^/s except in February for RCP8.5. The highest streamflow is predicted during August to December for both future periods under RCP8.5. The seasonal changes in streamflow range between –2.8% and –4.3% during the off season, and between 0% (nil) and –3.8% during the main season. The assessment of the impacts of climatic variabilities on the available water resources is necessary to identify adaptation strategies. It is supposed that such assessment on the Kurau River Basin under changing climate would improve operation policy for the Bukit Merah reservoir located at downstream of the basin. Thus, the predicted streamflow of the basin would be of importance to quantify potential impacts of climate change on the Bukit Merah reservoir and to determine the best possible operational strategies for irrigation release.

## Introduction

The variability in water resources is projected to increase with climate change and raise the risk of disasters; it will affect water and food security and economic growth. So, actions for managing water should be focused on climate change with an emphasis on basin-scale hydrological management techniques^1^. Human-induced climate change is continuously altering the hydrological systems and, consequently, affecting water resources with implications for many sectors, such as agriculture, forestry, fisheries and inland navigation^2^. Asia is the largest rice producer and consumer after wheat, and the demand of rice has been predicted to increase by 50% by 2050^3,4^. In Malaysia, rice is usually cultivated under irrigation to safeguard continuous and sustainable production. Water demand with double-cropping rice cultivation is quite high, consuming almost 80% of Malaysia’s water resources with constant supply^2,5^. Most rice farming systems in the country depend primarily on river water, which is often inadequate, especially during the off season (February to July). As a result, problems are encountered in allocating irrigation water to rice fields. The Kurau River Basin is the main source of water for the large Kerian Irrigation Scheme in the basin. To cope with the problems of water shortage, some rice schemes integrate reservoir with river headwork to satisfy irrigation water demands. The hydrological cycles of the river basin under climate change conditions are therefore very important in planning and controlling the irrigation scheme’s potential water demands.

The temporal and spatial variations of rainfall cause uncertainty in water availability for irrigation^6,7^. In Malaysia, the number of wet/rainy days is projected to decrease^8^. Most farmers are already facing climatic vulnerability based on their common perceptions without knowing the actual climate change issues^9^. But, selecting the effective adaptation strategies needs proper understanding of how the global climate change will affect local environment. So, the first step towards climate change impact assessment in hydrological systems is understanding the information extracted from global climate models (GCMs)^10^. The GCMs are so far the most advanced tools for simulating the response of climate system to increasing greenhouse gas concentrations. However, because of the GCMs’ relatively coarse spatial scale, their outputs cannot be directly applied to impact assessment models at the local to regional scale. GCMs’ outputs are normally downscaled to the resolution of observations used for inputs to various hydrological and crop-growth models (e.g., Variable Infiltration Capacity Model (VIC), HEC-HMS, TOPMODEL, MIKESHE, CropWat 8.0, AquaCrop, DSSAT, etc.). The choice of the downscaling method depends on purpose of the study, availability of station data, scale of needed data and size of study area.

Currently, simulation results of regional climate model, especially for the latest generation of GCMs CMIP5 using RCP scenarios, are not available for Malaysia. Instead, the delta or change factor approach^11–13^ is typically used to generate inputs to the Soil and Water Assessment Tool, SWAT, to assess global climate change’s hydrological impacts on the Kurau River Basin. Previous works^14^ described application of the SWAT model in Malaysia and summarised its global application. The objective of this study was to assess the projected changes in monthly flow of the Kurau River Basin by: (i) evaluating effectiveness of SWAT model in predicting monthly streamflow in the Kurau River Basin using station observations, (ii) downscaling multiple GCMs using the delta or change factor approach under Climate-smart DSS for the Kerian Irrigation Scheme, and (iii) investigating future changes in streamflow of the basin under different scenarios.

## Materials and Methods

### Study area

**Figure 1:**
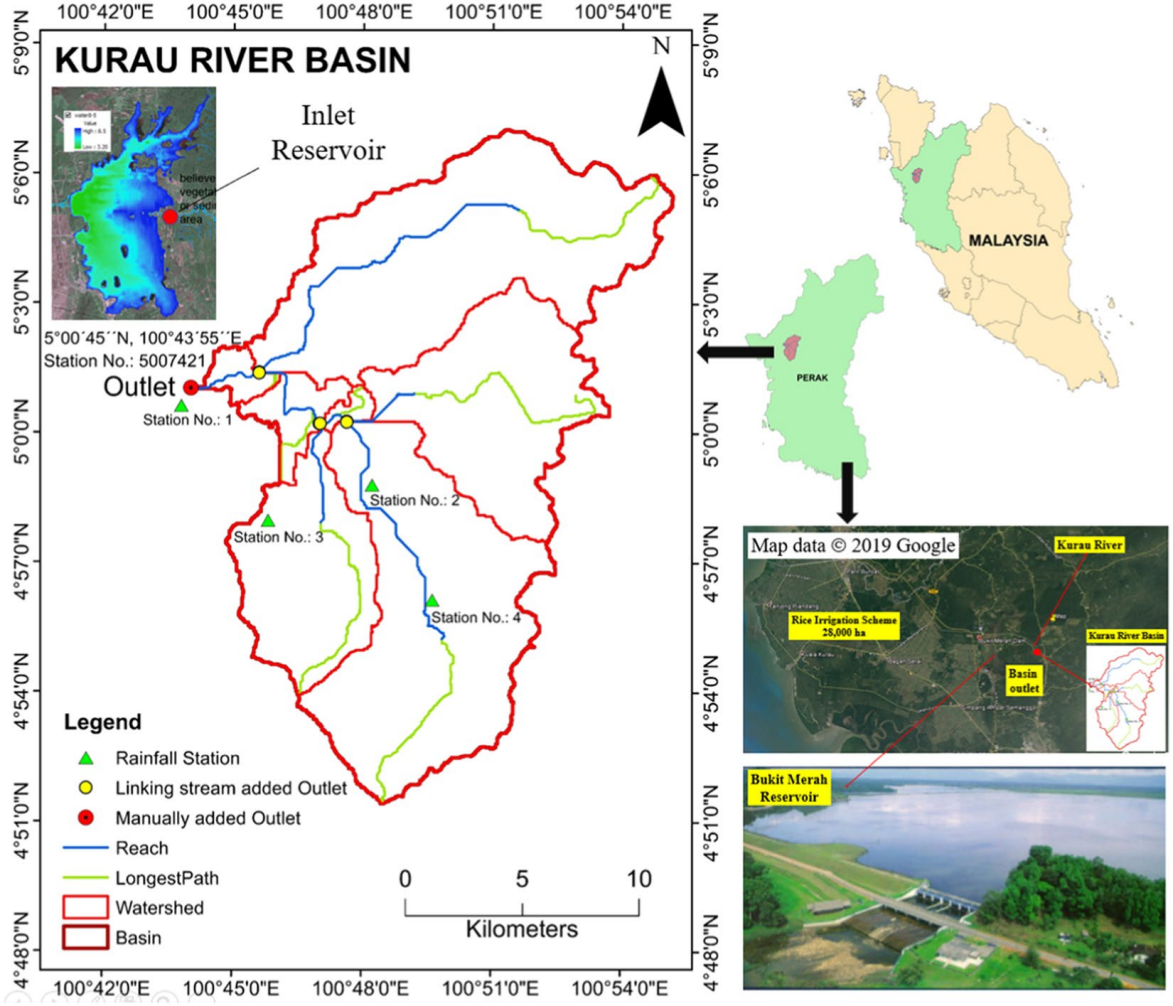
Location of the Kurau River Basin in the Perak state of Malaysia (*Adapted from Google Map & DID Kerian Irrigation Scheme*).

The Kurau River Basin (Fig. 1) has an area of 322 km^2^ and is located at the northern part of Perak in Peninsular Malaysia (4°51′–5°10′N and 100°43′–101°55′E). The basin is a dominant part of the Bukit Merah Reservoir catchment with two main rivers, the Ara and Kurau^15^. The area has a humid tropical climate characterized by southwest monsoon during February to July and northwest monsoon during August to January. The average total annual rainfall of the area is 2500 mm, which is concentrated mostly between April and October, and the average temperature is 27 °C. There are four meteorological stations, which are located at Pondok Tanjung (5007020, Station 1), Ladang Norseman (4907019, Station 2), Pusat Kesihatan Kecil of Batu Kurau (4908018, Station 3) and Ibu Bekalan Sempeneh of Batu Kurau (4908013, Station 4). All climatic data are recorded at these meteorological stations. The Bukit Merah Reservoir is the main water source for irrigation to the Kerian Irrigation Scheme. Streamflow from the basin outlet is measured at Pondok Tanjung station (5007421) located at the Kurau River.

### Study framework

**Figure 2:**
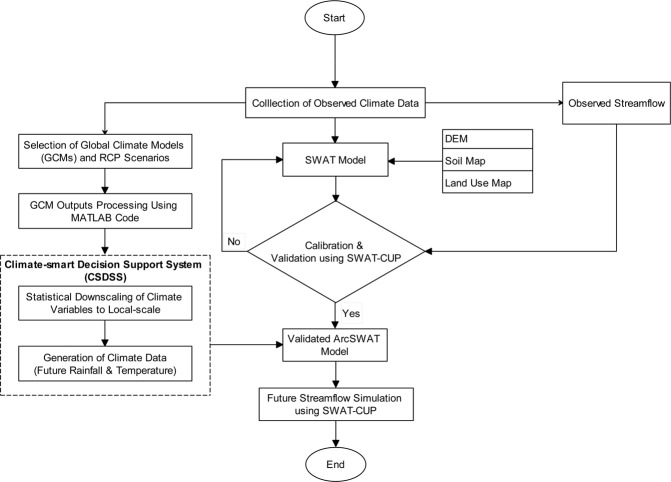
Study framework for assessment of climate change impacts on future streamflow.

The methodological framework of the study is schematically illustrated in Fig. 2. It encompassed 3 major features: (i) MATLAB program for processing and analysing large climate data set from GCMs, (ii) downscaling rainfall and climate variables from an ensemble of GCMs using Climate-smart DSS^16^, and (iii) application of ArcSWAT model to simulate hydrologic response of the Kurau River Basin to climate change impacts.

### Input data sets

**Figure 3:**
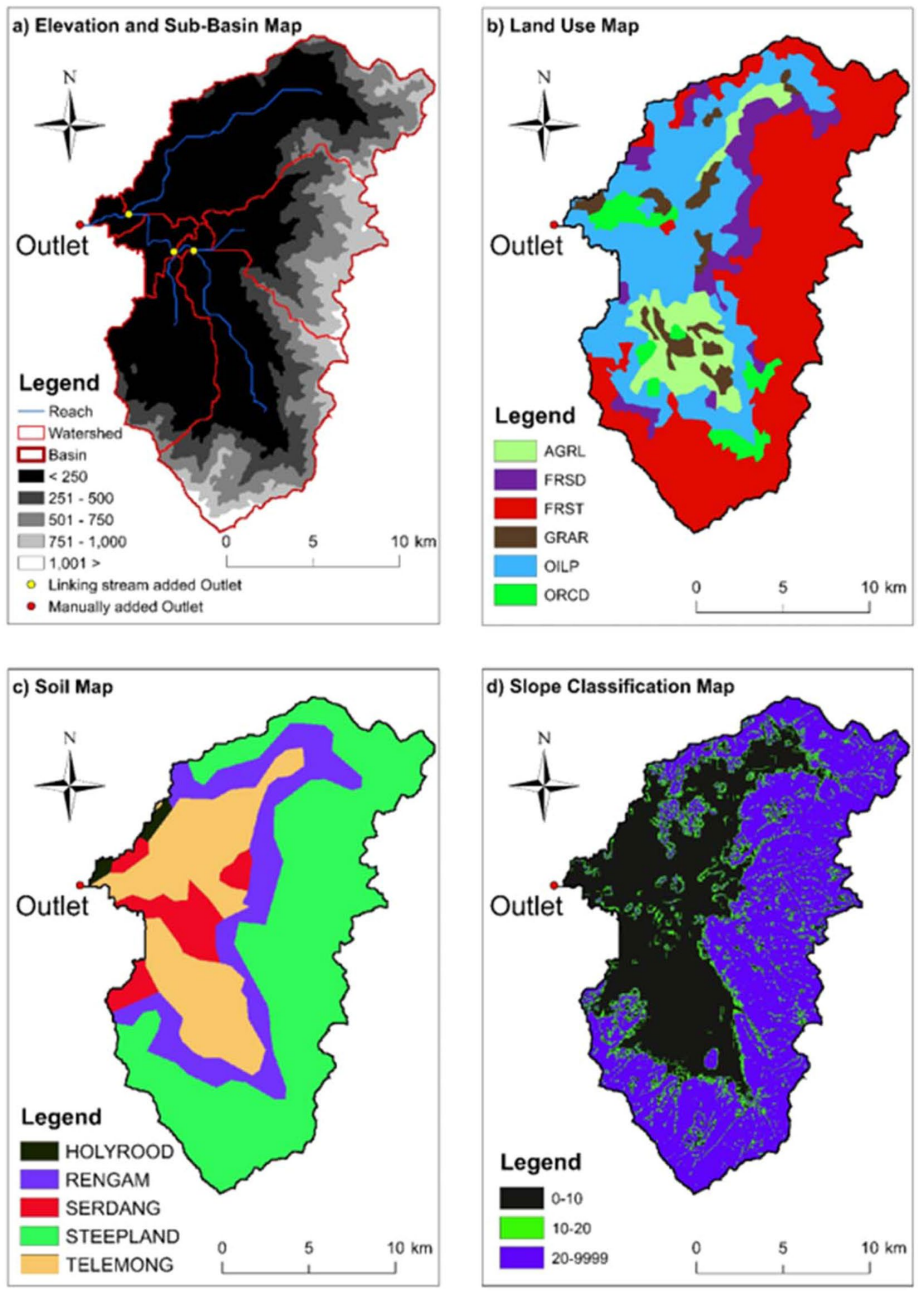
Input spatial data for ArcSWAT model for the Kurau River Basin: (**a**) digital elevation model of 30-m resolution, (**b**) reclassified land use data set, (**c**) soil data set, and (**d**) slope classification.

Table 1 summarizes daily climate and spatial data, including data-type and their sources that are needed to initialise hydrological simulation in SWAT. Spatial information used in the model includes watershed land use/land cover (LULC), soil map and Digital Elevation Model (DEM). The LULC and soil maps were obtained from the Department of Agriculture (DOA), Malaysia. The 30 m × 30 m DEM, in raster format with Shuttle Radar Topography Mission (SRTM) spatial local reference data Kertau-RSO-Malaya-meters, was obtained from Diva-GIS. The land of the study area is predominantly forest (46.8%) and oil palm (28.6%). The spatial data used for this study area is depicted in Fig. 3. Rainfall data was collected from the Department of Irrigation and Drainage (DID), and minimum and maximum temperatures were collected from the Malaysian Meteorological Department (MMD). Other necessary climate data were: relative humidity, solar radiation and wind speed. The climate data are the main driving factors for streamflow scenario analysis in SWAT. Historical daily streamflow records for the period from 1976 to 2005 were collected from DID for gauging station No. 5007421 located at the outlet of the Kurau River Basin (Fig. 1). The gauging station had enough flow records for our study. There were a few missing data in the record that were estimated as the long-term average for the station.

**Table 1:** Summary of input data set, including data-type and their sources, for river flow simulation.

Data	Source	Description
Digital Elevation Model (DEM)	DIVA-GIS (www.diva-gis.org)	Elevation, overland, channel slopes, boundary
Soil map	Department of Agriculture (DOA), Malaysia (www.doa.gov.my)	Soil classification and properties
Land use map	Department of Agriculture (DOA), Malaysia	Land use classification – cropland, forest, pastures, etc.
Climate data	Malaysian Meteorological Department (MMD), Malaysia	Daily maximum and minimum temperatures (1976–2005)
Streamflow	Department of Irrigation and Drainage (DID), Malaysia (www.water.gov.my)	Daily rainfall, daily streamflow (1975–2005)

Simulations of 10 CMIP5 GCMs obtained from the Climate Model Diagnosis and Inter-comparison (PCMDI) were used to generate future climate projections for the Kurau River Basin. Multiple GCMs (Table 2) were selected to span the range of scientific uncertainty in simulating the response of the climate system to increasing human emissions^17^. Selection of the GCMs was based on: (i) availability of climate variables in the GCM archives and (ii) availability of variables at daily time-step for baseline and future periods. Future simulations were done based on three scenarios: a lower scenario (RCP4.5), a medium-high scenario (RCP6.0) and a higher scenario (RCP8.5); RCP2.6 scenario was not included in this study.

**Table 2:** List of 10 Global Climate Models (GCMs) used in this study.

Organization	GCM	Atmospheric resolution	
		Lat.	Lon.
Canadian Centre for Climate Modelling and Analysis	CanESM2	2.8	2.8
National Centre for Atmospheric Research	CCSM4	1.25	0.94
Centre National de Recherches Metrorologiques/Centre Europeen de Recherche et Formation Avancee en Calcul Scientifique	CNRM-CM5	1.40	1.40
Commonwealth Scientific and Industrial Research Organization in collaboration with Queensland Climate Change Centre of Excellence	CSIRO	1.80	1.80
NOAA Geophysical Fluid Dynamics Laboratory	GFDL-ESM2G	2.50	2.00
NOAA Geophysical Fluid Dynamics Laboratory	GFDL-ESM2M	2.50	2.00
Met Office Hadley Centre	HadGEM2-CC	1.88	1.25
Met Office Hadley Centre	HadGEM2-ES	1.88	1.25
Max-Planck-Institut für Meteorologie (Max Plank Institute for Methodology)	MPI-ESM-LR	1.88	1.87
Meteorological Research Institute	MRI-CGCM3	1.10	1.10

### Model setup

We used ArcSWAT 2012, a GIS-based version freely available through SWAT website. The DEM of the Kurau River Basin and a pre-defined digital stream network layer were loaded to ArcSWAT. Subsequently, land use map and soil map of the area were uploaded into SWAT and the watershed was delineated into sub-basins for topographic analysis. Lookup tables for land use and soil were generated from land use/land cover, LULC. Hydrologic Response Units (HRUs) were defined after discretization of the sub-basins. The HRUs analysed the lookup tables to recognize SWAT code for different categories of land use and soil in map format. The soil map was linked to soil database in SWAT-operating folder, which holds data of soils worldwide. The entire watershed was classified into three slope categories depending on watershed topography. For multiple HRU definitions, land usages, soils and slope categories that covered less than threshold level of sub-basin area were eliminated. Input files for daily weather generator data (WGEN-User) and rainfall from the weather stations (lookup tables) were prepared to run streamflow simulation at the outlet gauging station No.1 (No. 5007421). The SWAT model has the ability to generate missing hydrological data based on completed file of meteorological data sets. Finally, the simulated output flow for the Kurau River Basin was obtained.

### Model calibration and validation

Calibration and validation of the SWAT output were done by SWAT-CUP^18^. Model calibration is a process of choosing parameters that are best suited to local conditions to minimize uncertainty, while model validation is a process of demonstrating whether or not the model’s calibrated parameters can provide adequate and precise prediction based on projected purposes^19^. We utilized Sequential Uncertainty Fitting (SUFI-2) algorithm for model calibration and validation. SUFI-2 has the ability to handle several parameters, and it is a combination of calibration and uncertainty analysis to determine parameter uncertainties.

In this study, 30 years’ (1976–2005) monthly streamflow data were used for evaluation of the model. Calibration of the model was done using 18 years’ (1981–1998) data and validation was done using 7 years’ (1999–2005) data. For calibration and validation of the model, the time periods 1976–1980 and 1996–1998 were used as warm-up periods to eliminate initial bias. For a cross-check, we also calibrated the model using another 10 years’ (1981–1990) data set and validated it using another 15 years’ (1991–2005) data set. Fourteen parameters related to streamflow were selected for model calibration based on recommendations from SWAT-CUP manual. The initial ranges of the parameters were determined based on information from SWAT user guide. In order to capture the basin’s hydrological process, three parameter qualifiers (r_relative, a_absolute and v_replace) were adjusted. About 500 simulation runs were executed to calculate uncertainty and statistical properties, and also to generate new ranges of the parameters. The new final parameters, with the highest sensitivity values, were obtained and applied in the model validation without any further modifications.

### Assessment of model prediction

Coefficient of Determination (R^2^), Nash-Sutcliffe Efficiency (NSE) and Percent Bias (PBIAS) were used to test model efficiency. The association between simulated and observed streamflows is quantified by these indicators. NSE is commonly used in hydrological studies; it tests how well the simulated data plot matches 1:1 line against the observed data^20^. PBIAS tests the simulated data’s average tendency to become more or less than their observed counterparts^21^. The evaluation of model prediction can be classified satisfactory if both the R^2^ and NSE > 0.5 and PBIAS <±25. The various indicators for assessing the model’s performance are expressed as follows:

1 $${R}^{2}={\left(\frac{{\sum }_{i=1}^{n}({P}_{i}^{obs}-{P}_{i}^{mean})({P}_{i}^{sim}-{P}_{i}^{mean})}{{[{\sum }_{i=1}^{n}{({P}_{i}^{obs}-{P}_{i}^{mean})}^{2}{\sum }_{i=1}^{n}{({P}_{i}^{obs}-{P}_{i}^{sim})}^{2}]}^{0.5}}\right)}^{2}$$

2 $$NSE=1-\left[\frac{{\sum }_{i=1}^{n}{({P}_{i}^{obs}-{P}_{i}^{sim})}^{2}}{{\sum }_{i=1}^{n}{({P}_{i}^{obs}-{P}^{mean})}^{2}}\right]$$

3 $$PBIAS=\left[\frac{{\sum }_{i=1}^{n}({P}_{i}^{obs}-{P}_{i}^{sim})\times 100}{{\sum }_{i=1}^{n}({P}_{i}^{obs})}\right]$$

In Eqs. 1 to 3, ${P}_{i}^{obs}$ is observed flow, ${P}_{i}^{sim}$ is simulated flow, *P*^*mean*^ is mean of the observed flow, and *n* is total number of flow observations.

### Climate-smart DSS user-interface for downscaling GCMs

**Figure 4:**
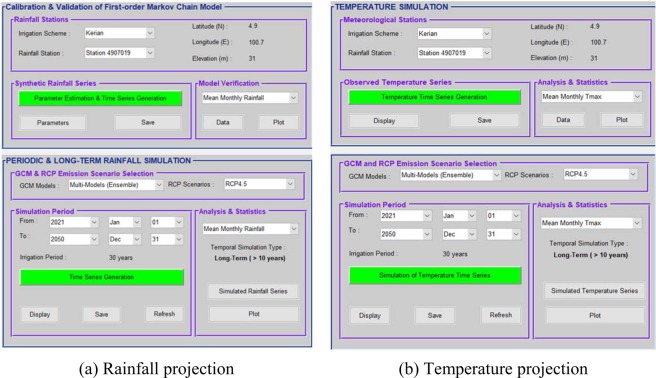
Dialog windows for simulating hydro-meteorological sequences using CSDSS.

The Climate-smart DSS (CSDSS) tool consists of two basic modules: (i) stochastic simulation and (ii) temperature simulation. The user requires selecting the station, variables of interest, combination of GCM-RCP and simulation period to run a rainfall simulation. First, the future monthly temperature and rainfall at a station is produced by perturbing the station using change factors from the projected mean changes simulated by the GCMs. The outputs can be generated as daily time series and long-term monthly time-scale and can be viewed from the “*Analysis and Statistics*” command button as tables and graphs (Fig. 4).

In this study, 1976–2005 was adopted as baseline period, and 2021–2050 (30 years) and 2051–2080 (30 years) were considered as two future periods. Simulation outputs can be obtained from each of the 10 GCMs or through multi-models’ projections based on the selected RCP scenario. Second, daily rainfall is generated from the scaled monthly values using a ‘Richardson-type’ weather generator, which is regarded as an acceptable tool in studying impacts of climate on a variety of systems, including ecosystem and risk assessment^22^. The weather generator consists of two types of rainfall modelling for any given time-scale those simulate both rainfall occurrence and rainfall amount separately. The sequence of rainfall occurrence is generated using first-order Markov chain model, while the rainfall amount is generated by applying gamma distribution. The gamma distribution is fitted to all days to be modelled as wet days, and a threshold value of 1 mm was considered for Malaysia due to high humidity condition^23,24^. Details of successful application of this approach can be found in the references herein^25–27^.

## Results and Discussion

### Climate-smart DSS Outputs

#### Projected future rainfall

**Figure 5:**
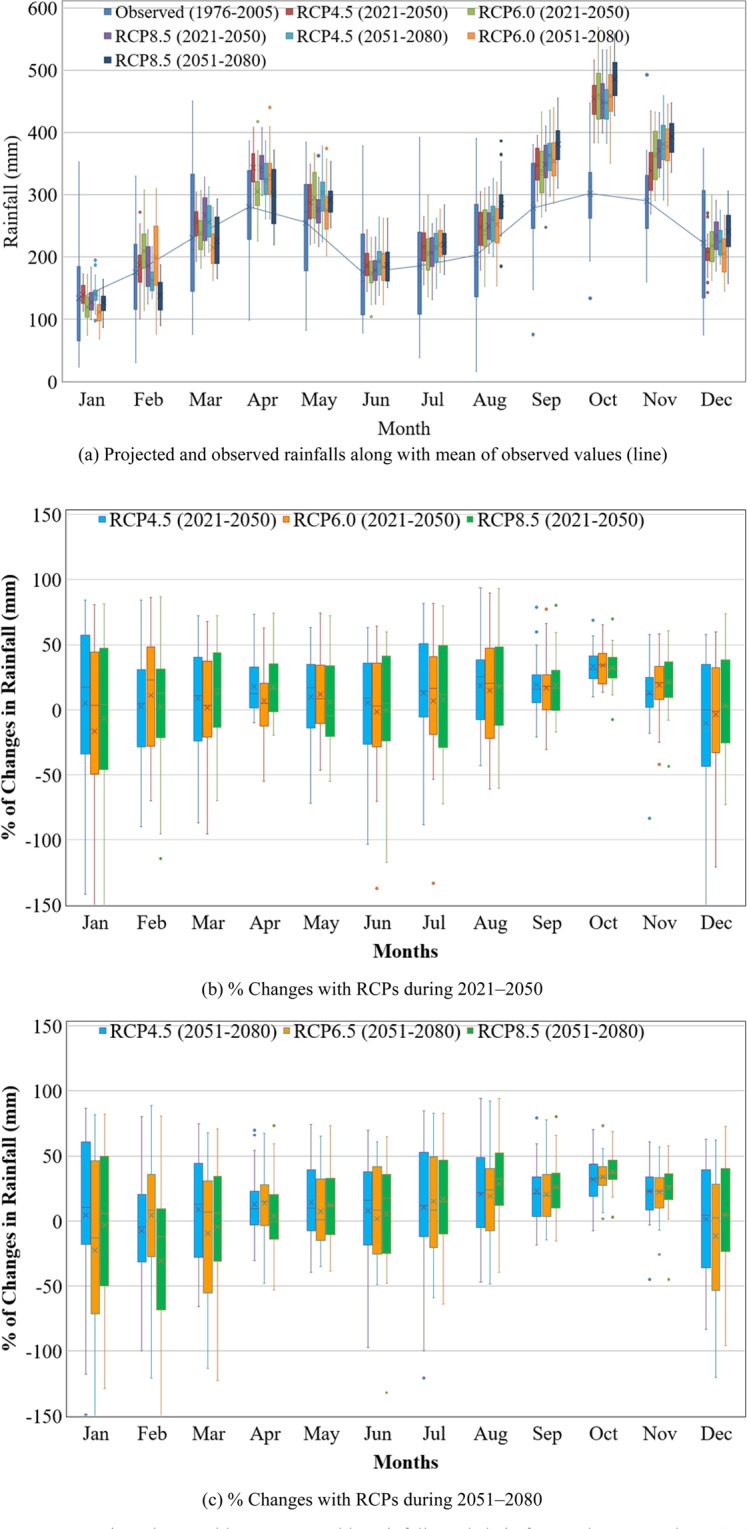
Projected ensemble mean monthly rainfalls and their future changes under RCP4.5, RCP6.0 and RCO8.5 scenarios for future periods of 2021–2050 and 2051–2080 with respect to the baseline period of 1976–2005. (**a**) Projected and observed rainfalls along with mean of observed values (line). (**b**) % Changes with RCPs during 2021–2050. (**c**) % Changes with RCPs during 2051–2080.

The projected mean monthly rainfalls, based on the ensemble of GCMs’ simulations and three RCPs (RCP4.5, RCP6.0 and RCP8.5) for two future periods of 2021–2050 and 2051–2080 with respect to the baseline period of 1976–2005, are illustrated in Fig. 5.

The three RCP scenarios reveal increasing trend of the future mean monthly rainfall during 2021–2050 and 2051–2080 periods except in January, February, March and June, which show decreasing trend in rainfall. In Malaysia, lower rainfall is observed usually in February–March and May–July than in the other months of the year. So, more dry days are expected to appear in these months in the future. In both future periods, the predicted mean monthly rainfalls show significant increasing trends in April, September, October and November. Greater increase in the mean monthly rainfall is predicted under RCP8.5 scenario compared to RCP4.5 and RCP6.0 scenarios during 2021–2050. The predicted monthly rainfall was compared with the recorded rainfall and the observed deviations were analysed. The box and whisker plots in Fig. 5(b,c) illustrate the multi-models’ ensemble of the projected changes by 10 GCMs. Although few GCMs project negative changes, most scenarios project increasing trend of the average monthly rainfall except in January, February and March. It is clearly evident from the projection that the monthly rainfall pattern will change in the future compared to the baseline period of 1976–2005. The annual rainfall pattern, however, will remain invariable. The future projection reveals decreasing trends in rainfall in only a few months but increasing trends in most of the months of the year. Therefore, the impacts of climate change will affect agricultural water management by bringing longer dry spells in a few months and excess rainfall in most months of the year. This climate change impact will alter the hydrologic response of the watershed and affect inflows to the reservoirs, especially the downstream reservoirs.

#### Projected changes in temperatures

**Figure 6:**
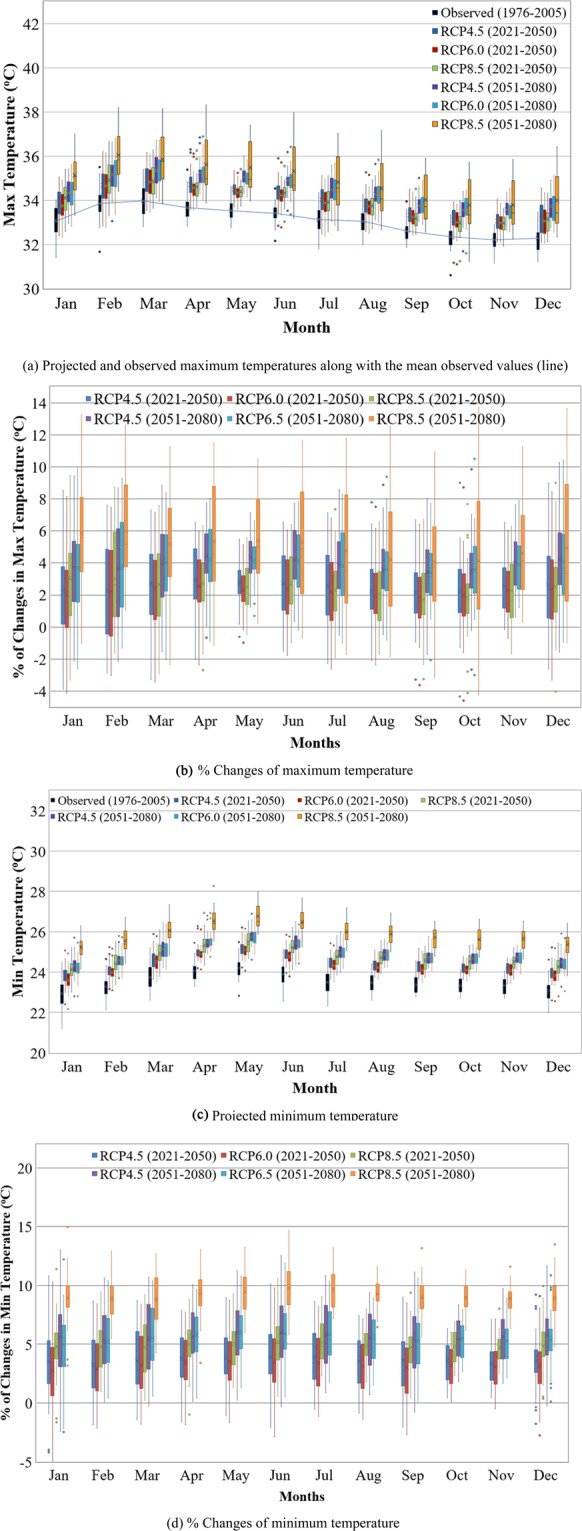
Mean monthly changes in maximum and minimum temperatures for the future periods of 2021–2050 and 2051–2080 under RCP4.5, RCP6.0 and RCP8.5 scenarios. (**a**) Projected and observed maximum temperatures along with the mean observed values (line). (**b**) % Changes of maximum temperature. (**c**) Projected minimum temperature. (**d**) % Changes of minimum temperature.

RCP4.5, RCP6.0 and RCP8.5 scenarios reveal increase in both the maximum and minimum temperatures throughout the two future periods of 2021–2050 and 2051–2080 (Fig. 6). Under these RCP scenarios, the mean maximum temperature is projected to increase by 1.0 °C during 2021–2050 and 1.5 °C during 2051–2080 compared to the baseline period. The minimum temperature is projected to increase by 1.0 °C and 1.6 °C for the corresponding future periods.

As expected, the largest change in both the maximum and minimum temperatures was projected for both future periods under the highest RCP (RCP8.5) scenario, with greater change obtained in the later period. However, difference in temperature change (both maximum and minimum temperatures) is evident among RCP4.5, RCP6.0 and RCP6.0 scenarios.

### Impacts of future climate change on Kurau river basin

#### SWAT model calibration and validation results

Historical rainfall data of three meteorological stations and temperature data of one meteorological station were used to setup SWAT model for the baseline period. Sensitivity and uncertainty analyses were executed using the most effective number of model parameters that had more significant influences in simulating streamflow within the SWAT-CUP program. Fourteen (14) model parameters influencing the streamflow behaviour were selected (Table 3) for the sensitivity and uncertainty analyses.

**Table 3:** Changes in SWAT-parameters during calibration stage.

Rank	Parameter Name	Parameter Description	Parameter Range		Calibrated Value
			Min	Max	
1	ALPHA_BF.gw	Baseflow alpha factor (days)	0	0.5	0.27
2	CH_N2.rte	Manning’s value for main channel	−0.01	0.11	0.06
3	REVAPMN.gw	Threshold depth of water in the shallow aquifer for “revap” to occur (mm)	100	350	278.75
4	ESCO.hru	Soil evaporation compensation factor	0	1	0.22
5	SOL_K.sol	Saturated hydraulic conductivity	4000	6000	4438.00
6	CH_K2.rte	Effective hydraulic conductivity in main channel alluvium	−0.01	500	195.49
7	EPCO.hru	Plant uptake compensation factor	0	1	0.04
8	CN2.mgt	SCS runoff curve number	−0.6	3	−0.53
9	GW_REVAP.gw	Groundwater “revap” coefficient	0.02	0.2	0.02
10	GWQMN.gw	Threshold water depth in shallow aquifer required for return flow to occur (mm)	1000	5000	4180.00
11	GW_DELAY.gw	Groundwater delay (days)	100	500	234.00
12	SLSUBBSN.hru	Average slope range	50	150	125.30
13	CANMX.hru	Maximum canopy storage	50	100	92.85
14	SOL_AWC.sol	Available water capacity of the soil layer	0	1	0.43

**Figure 7:**
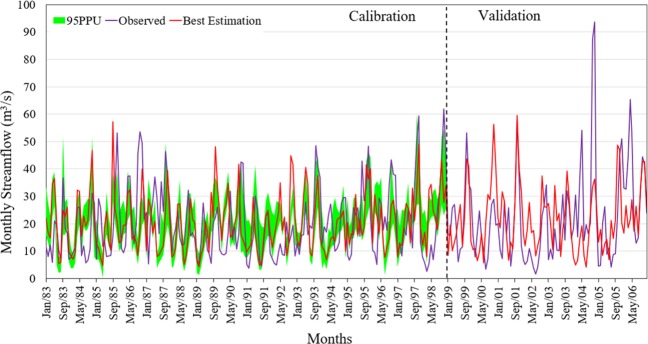
Comparison of the observed and simulated streamflows for calibration (1981–1998) and validation (1999–2005) periods.

Results in the calibration period are relatively better than that in the validation period. The performance indices, R^2^ and NSE (Eqs. 1 and 2), rarely become higher in validation than in calibration period since the parameters are optimized during the calibration period^11,28,29^. Figure 7 depicts that the observed and simulated streamflows during calibration (with 18 years’ data) and validation (with 7 years’ data) agree fairly well. Calibrated tests are usually satisfactory with R^2^, NSE and PBIAS (Eqs. 1–3) of 0.65, 0.65 and −3.0, respectively. The corresponding values of the indices for validation period are 0.60, 0.59 and −4.6. When the model was calibrated using 10 years’ data and validated using 15 years’ data, R^2^, NSE and PBIAS became 0.61, 0.60 and −2.7, respectively during calibration and 0.72, 0.60 and −2.7, respectively during validation, indicating some improvement in the validation results. The detail results of this second-stage calibration and verification of the model are provided as supplementary materials. The model was able to capture some of the peak flow events both during calibration and validation. It however under-predicted the flow in September to November during which the rainfall was very high. This observation is in agreement with the previous findings^30^ that reported inability of SWAT to simulate extreme events well. It is evident that the validation result for the simulated monthly streamflow greatly deviated from the observed values, especially in August 2003 and December 2005. Nevertheless, the overall model performance is acceptable and, so, the model is suitable to simulate streamflow as proposed^31^.

#### Projected future streamflow

**Figure 8:**
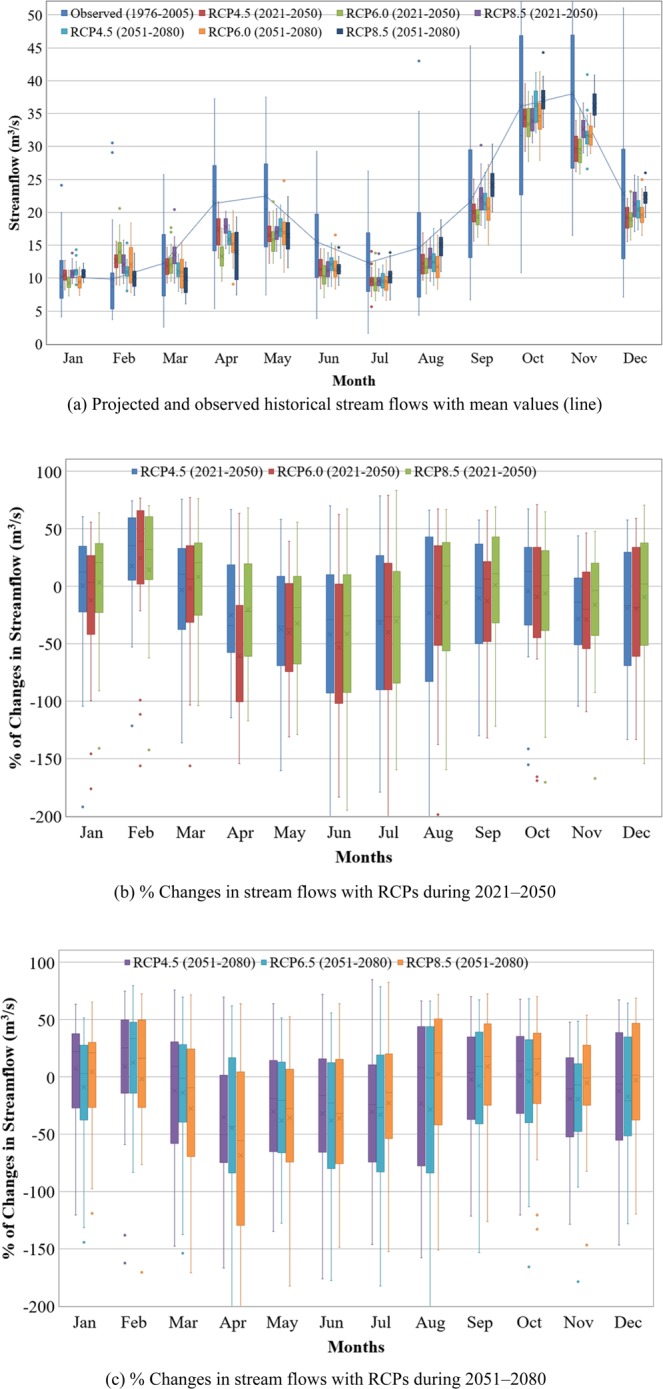
Projected ensemble mean monthly streamflows and their future changes under RCP4.5, RCP6.0 and RCO8.5 scenarios for future periods of 2021–2050 and 2051–2080 with respect to the baseline period of 1976–2005. (**a**) Projected and observed historical stream flows with mean values (line). (**b**) % Changes in stream flows with RCPs during 2021–2050. (**c**) % Changes in stream flows with RCPs during 2051–2080.

Figure 8 illustrates the average seasonal future changes in streamflow during the northwest monsoon (August to January, main season) and southwest monsoon (February to July, off season) relative to the baseline period. During the main season of both future periods (2021–2050 and 2051–2080), the seasonal streamflow is projected to decrease for RCP4.5 and RCP6.0 scenarios; there will be only minimal change in streamflow during 2051–2080 for RCP8.5 scenario. The seasonal streamflow during the off season is projected to decrease in both future periods for all three RCP scenarios. During 2021–2050 period in the off season, the highest decrease is predicted for RCP6.0 scenario followed by RCP4.5 and RCP8.5 scenario, while the highest decrease in streamflow during 2051–2080 period is predicted for RCP8.5 followed by RCP6.0 and RCP4.5 scenario. The impacts of climate change will therefore alter the hydrologic responses of the Kurau River Basin as reflected by the chnages in streamflow in the future periods of 2021–2050 and 2051–2080.

**Figure 9:**
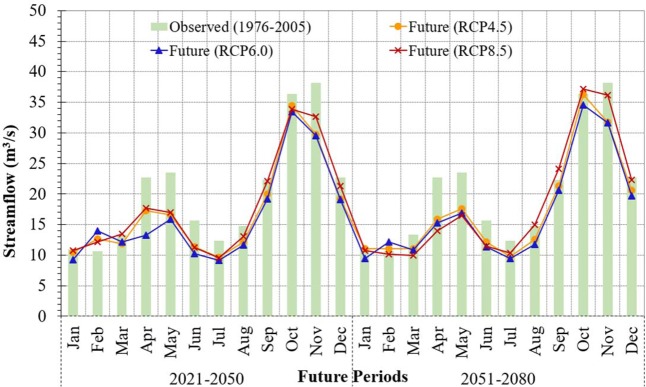
Projected mean monthly streamflows predicted by multi-models under RCP4.5, RCP6.0 and RCP8.5 for two future periods (2021–2050 and 2051–2080) compared to baseline period (1976–2005).

Generally, the projected streamflow of ensembles of multi-models decreases for the selected RCP scenarios with respect to baseline period (Fig. 9). The predicted future streamflow witnesses a clear decrease from 8 m^3^/s to 2 m^3^/s during May to July for the three RCP scenarios and two future periods. In January, the streamflow does not change much; the reduction in streamflow, compared to the baseline period, remains below 2 m^3^/s. While, the future streamflow in February is predicted to increase by 1.5 m^3^/s except for RCP8.5 during 2051–2080, when there is a decrease in streamflow by less than 0.5 m^3^/s. The future streamflow during March and April is predicted to be higher during 2021–2050 but lower during 2051–2080 for RCP8.5. Higher streamflow is predicted during August to December for RCP8.5 compared to RCP4.5 and RCP6.0 scenarios for both future periods. The ranges of streamflow in the corresponding months for the three RCPs are 11–15 m^3^/s, 19–25 m^3^/s, 33–38 m^3^/s, 29–37 m^3^/s and 19–23 m^3^/s. The projected streamflows for the two future periods under the three RCPs are lower than those for the baseline period except in February. These observations reveal that temperature plays a key role on the hydrologic response in the Kurau River Basin. The streamflow projection is in contrast with the projected rainfall patterns due to the fact that the increasing temperature will boost evapotranspiration in the basin and consequently reduce streamflow in the future.

**Figure 10:**
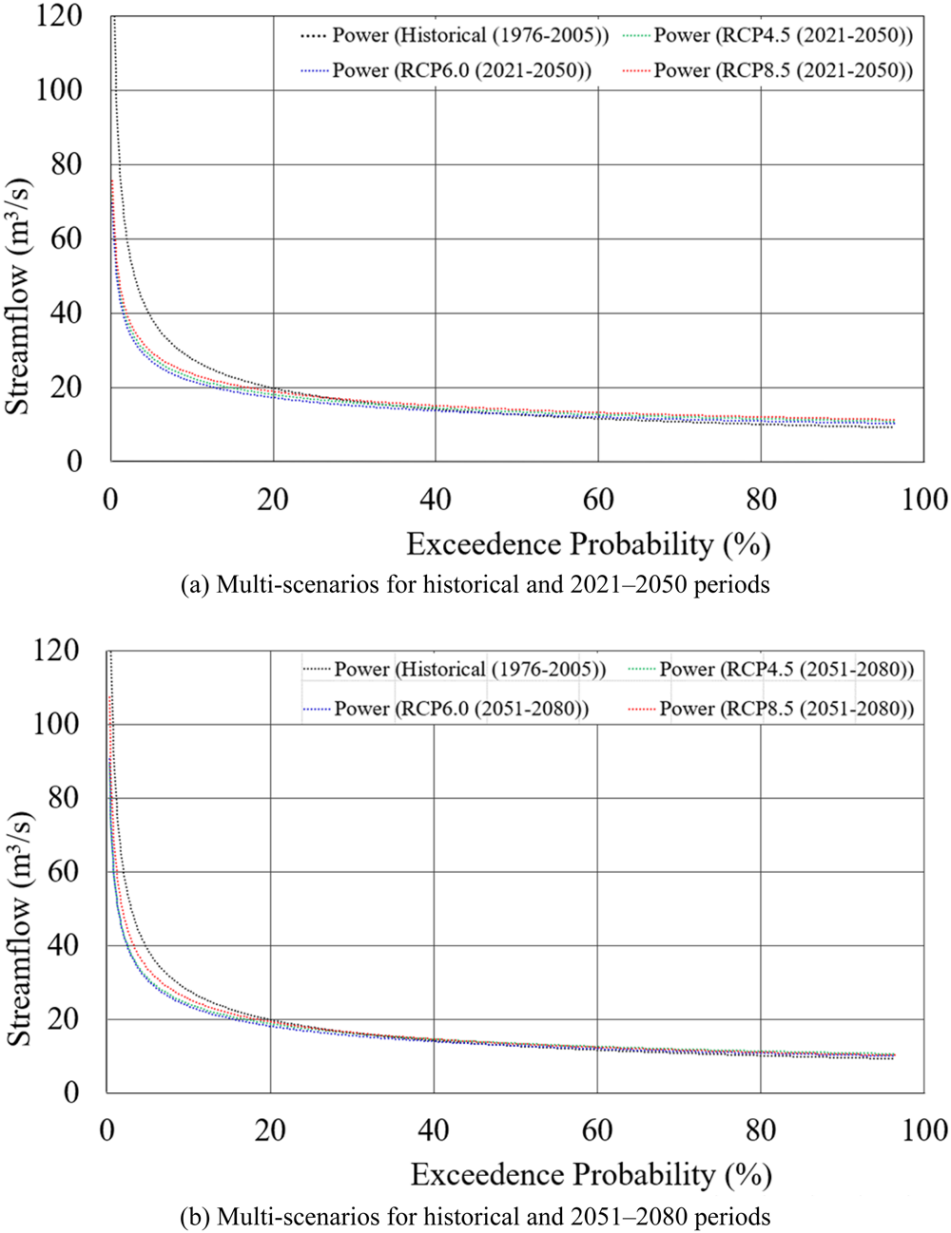
Cumulative frequency distribution curves of the projected ensemble mean monthly streamflow at the Kurau River Basin for the historical and future periods. (**a**) Multi-scenarios for historical and 2021–2050 periods. (**b**) Multi-scenarios for historical and 2051–2080 periods.

Figure 10 illustrates cumulative frequency distribution for the ensemble mean monthly projected streamflows of the basin. The projected outputs show that streamflow is more likely to decrease for all three RCP scenarios in the future. Although there is a decreasing trend in streamflow in both future time periods, in general, there are no much differences among the three RCPs. Therefore, based on the projected streamflow, it may be confirmed that climate change will have impacts on hydrologic response of the Kurau River Basin. With increasing probability, the streamflow will decrease due to climate change in the future and, consequently, the balance of water resource structure will also change. It will certainly and negatively affect the inflow to and release from the reservoir storage to meet the irrigation demand.

#### Uncertainty in streamflow prediction

**Figure 11:**
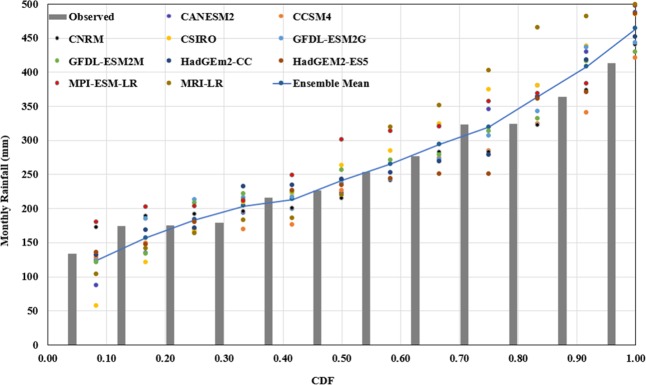
Variability among downscaled individual GCM and ensemble of 10 GCMs, and 3 RCPs with recorded monthly rainfall.

The probable reasons for poor performance of the model in some cases could be attributed to rainfall. Table 4 lists the determination coefficient, R^2^, which defines predictable percentage of variance in the observed monthly rainfall by downscaling of GCMs data. The GCM-derived monthly rainfall of ensemble GCMs produced large R^2^ values for the RCP scenarios: 0.85 for RCP4.5, 0.95 for RCP6.0 and 0.96 for RCP8.5. It thus implies that downscaled GCMs data are acceptable for the effect analysis. The plot of Cumulative Density Function (CDF) describes variability of the reduced precipitation of individual GCM and ensemble GCM, and observed and simulated precipitations (Fig. 11).

**Table 4:** Comparison of R^2^ for GCM simulations with the observed monthly mean rainfall.

GCMs/RCPs	CANESM2	CNRM	CCSM4	CSIRO	GFDL-ESM2G	GFDL-ESM2M	HadGEM2-CC	HadGEM2-ES	MPI-ESM-LR	MRI-CGCM3	Ensemble
RCP45	0.909	0.819	X	0.718	0.936	0.851	0.838	0.894	X	0.811	**0.974**
RCP60	0.952	0.909	X	0.615	0.847	0.899	X	0.835	X	0.900	**0.952**
RCP85	0.750	0.882	0.827	0.823	0.891	0.826	0.790	0.842	0.937	0.870	**0.967**

Different model indices, computed using monthly streamflow data, are listed in Table 5. During model calibration period (1981 to 1998), the p-factor was 0.72 and r-factor was 1.03. The value of the corresponding factor was 0.71 and 1.18 during model validation period (1999 to 2005). Thus, the relatively lower values of the factors during calibration period reveal that the model uncertainty is less in that period. It was found that the recorded peak streamflows were not included in the 95% prediction uncertainty band in the years 1992 and 1995 during calibration and in the year 2003 during validation. This was due to that the SWAT model cannot replicate extreme events and forecasts^30,32^. The parameter uncertainties are acceptable when both the p- and r-factors reach the desired low limits or reveal smaller prediction uncertainty. The values of R^2^ and NSE obtained during calibration were both 0.70. The values of R^2^ and NSE obtained during validation were 0.65 and 0.63, respectively, which suggest that the model may be accepted for the Kurau River Basin. The NSE value greater than 0.75 indicates that the simulation results are good, and when NSE is within 0.36 and 0.75, the simulation results are satisfactory^31,33^. Thus, our results of the simulation are good and the outcomes of the simulation results are satisfactory.

**Table 5:** Statistical indices for evaluation of monthly streamflow calibration (1981–1990) and validation (1991–2005).

Model performance indices	Calibration	Validation
Coefficient of determination (R^2^)	0.70	0.65
Nash-Sutcliffe Efficiency (NSE)	0.70	0.63
p-factor	0.72	0.71
r-factor	1.03	1.18

## Conclusions

The impact of climate change on streamflow of the Kurau River Basin in Malaysia was investigated by SWAT hydrological model using climate forecasts from 10 GCMs under RCP4.5, RCP6.0 and RCP8.5 scenarios. Multi-climate models simulated local climate variables from large-scale multi-climate models, and a DSS simulated long-term rainfall and temperature that were input to SWAT. The hydrological model successfully simulated monthly streamflow of the Kurau River Basin, with R^2^, NSE and PBIAS of 0.65, 0.65 and –3.0, respectively for calibration and 0.60, 0.59 and –4.6, respectively for validation period. The multi-model projections reveal 18% and 21% decrease in monthly rainfall in the Kurau River Basin under RCP8.5 during January to March of 2021–2050 and 2051–2080 period, respectively. The projected highest increase (27%) in monthly rainfall for the same scenario and periods was in August. The monthly temperature (both maximum and minimum) shows increasing trend throughout the future period (2021–2080) for all RCP scenarios, with periodic and seasonal variations. The temporal patterns show that the Kurau River Basin will experience high temperatures under RCP8.5 scenario in the future, particularly during 2051–2080 period.

The projected streamflow of the basin decreases for 2021–2050 and 2051–2080 periods compared to the baseline period of 1976–2005 under all RCP scenarios. However, based on our analysis, the streamflow volume in the study area should not experience any dramatic change in the future. The seasonal changes in streamflow range between –2.8% and –4.3% during the off season and 0% and –3.8% during the main season. It is noted that, due to uncertainty in the GCMs’ output and climate change scenarios, the future streamflow cannot be projected very precisely. To conclude, the general results of the study should be applied and incorporated to assess irrigation water demand in paddy scheme to cope with resilience of the Bukit Merah Reservoir. The results are expected to be used in assessing water demand of the irrigation scheme and to simulate optimum reservoir operation policy for the best water management practices under the impact of future climate change. Higher order stochastic rainfall generation can be employed to simulate the extreme hydroclimatic events in the basin. Furthermore, the outcomes of this study can serve as a reference through which climate modelers, climatologists and statisticians can work more closely. Climate modelers and researchers can carry out different downscaling using different statistical distributions and statistical models through their expert judgment. Moreover, we hope this work may foster more prudent analysis that incorporates scientific knowledge of climate modeling at regional and global scales. Because of only 30 years’ monthly data, we utilized 18 years’ data for calibration and 7 years’ data for validation of the model; the remaining 5 years’ data were used as warm-up period for calibration and validation of the model. Coordinated Regional Downscaling Experiment (CORDEX) for high resolution (5 km × 5 km) RCM climate projection for the southeast Asia and CMIP6 data are recommended for future assessment of the climate change impacts on streamflow at this basin with longer validation period.
